# Letter from G. Evans

**Published:** 1845-12

**Authors:** 


### ARTICLE VI.

### *Letter from* G. Evans.

*Social Circle, Ga. Nov. 20th*, 1845.

Sir :—Inclosed you will find a history of a case of rapid appearance of teeth (in the course of twenty-four hours) in sockets from which others had been extracted, and of fungous excrescence, &c., which occurred in a child of fours years and seven months of age. You will also find twelve of the teeth enclosed, the other twelve mouldered to dust, this occurred in Caswell County, North Carolina, some twelve years since. As I am only a student of dentistry, I send the history of the case, and the teeth to you, with a request that it be reported in the American Journal of Dental Science, with such remarks as you may deem proper to make, if you should think the case worthy of notice. Yours, &c.

JOHN H. GRAVES.

To Dr. C. A. Harris.

*Caswell County, N. C., Oct. 10th*, 1845.

Dear Sir :—I did not receive your letter of the 12th of August, till yesterday. We now proceed to answer your request, to the best of our recollection, as the case was not committed to writing at the time. The child was four years and seven months old when he first complained. Well and healthy before, was restless of nights a while before we knew any thing was the matter with him. One morning he came to his mother and said he had a sore in his mouth : she looked at it, and thought it was a gumboil. It continued to grow, and a few days after, Dr. John Comer was called ; he came, and he called the place a fungus. Two of his jaw teeth had become loose ; the doctor thought it advisable to extract them, and did so. This was on Friday, and the next day week I called the doctor again. By this time it appeared in both jaws: he then extracted several more, they being loose, and continued to get so till all his jaw teeth were extracted, from his eye teeth back, done at different times as they become loose. Twelve in number taken out, which had firm roots, and they were not decayed, no complaint of toothache, as they were extracted; others would appear, sometimes in twenty-four hours, which I extracted with my fingers, as the roots were soft or fleshy, as you may see by those I send you, the same number as the first. All his fore teeth remained firm and tight till his death. This all took place in one month from the day the doctor was first called to the time he died: he became very much swelled in body; he lost his flesh but still kept hearty to eat such as he could eat without teeth; he had always been a very healthy child. By examining those teeth I send, you find seven which are of the first set, the other five are of the last, the balance of the twenty-four, that was taken out, mouldered away to dust. The suffering he bore is past describing. The doctor operated two or three times on his jaw, but all to no effect.

Your friend, &c.

GOODWIN EVANS.

To Maj. John H. Graves.

P. S. I was a student of medicine at the time the case given by Mr. Evans occurred, and as far as I recollect, it is pretty correctly described; one important fact, however, which he has omitted, I will mention, that is, after all the teeth were extracted he brought him to Dr. Comer, where I was prosecuting the study of medicine, and I saw the doctor remove large fungous excrescences that had sprung up out of the jaw bone, when the teeth were extracted. This was frequently repeated afterwards, all however to no effect, as they continued to return as long as he lived, notwithstanding the actual cautery was frequently applied.

N. M. Roan.

Note.—The only thing mysterious or difficult of explanation in the case, here described, is, that so young a child should have been the subject of a fungous excrescence of the jaws. Eight of the teeth which were removed were the temporary molares, eight the bicuspides, whose crowns were only partially ossified, and the other eight, the first and second permanent molares, the last but partially ossified. The temporary teeth and permanent molares are in our possession. The fungous excrescence, as would seem, originated from the cells of the maxillary bones or rather alveolar border, above and beneath the teeth, which, by its growth, were removed from their sockets and brought to the surface of the gums.—*Balt. Ed.*

